# Selective Laser Trabeculoplasty: A Clinical Review

**DOI:** 10.5005/jp-journals-10008-1139

**Published:** 2013-05-09

**Authors:** Skaat Alon

**Affiliations:** Goldschleger Eye Institute, Sheba Medical Center, Tel Aviv University, Tel Hashomer, Israel; Glaucoma Clinical Fellow, New York Eye and Ear Infirmary, New York, USA

**Keywords:** Selective laser trabeculoplasty, Glaucoma, Trabecular meshwork.

## Abstract

Selective laser trabeculoplasty (SLT) is a safe and effective treatment modality for lowering the intraocular pressure in patients with glaucoma. It achieves its results by selective absorption of energy in the trabecular pigmented cells, sparing adjacent cells and tissues from thermal damage, with minimal morphological tissue alteration following treatment. On the basis of the peer-reviewed medical literature, SLT is efficacious in lowering IOP, as initial treatment or when medical therapy is insufficient in all types of open-angle glaucoma in all races. SLT achieves intraocular pressure reduction similar to that of argon laser trabeculoplasty but without the tissue destruction and side effects. Observed side effects following SLT were almost uniformly transient and minor. We review highlights of recently published studies on the mechanisms and clinical outcome of SLT in order to address frequently raised issues pertinent to SLT in the clinical practice.

**Key messages:** Selective laser trabeculoplasty is a safe and effective treatment modality for lowering the intraocular pressure in patients with glaucoma.

**How to cite this article:** Alon S. Selective Laser Trabeculoplasty: A Clinical Review. J Current Glau Prac 2013; 7(2):58-65.

## INTRODUCTION

Lowering the patient's intraocular pressure (IOP) is the mainstay of glaucoma treatment, in an attempt to arrest the characteristic progressive optic neuropathy and prevent irreversible visual field loss.^1^ This goal may be achieved either by medical, laser or surgical modalities.

Laser trabeculoplasty (LTP) has been used to lower IOP for 40 years, following the landmark publication of Wise and Witter.^2^ This procedure was originally performed using the then-common argon laser (major laser wavelengths at 488 and 514 nm), and so termed argon laser trabeculoplasty (ALT).

Several other lasers, including krypton (647.1 or 568.2 nm),^3^ diode (810 nm),^4^ and frequency-doubled Nd:YAG (532 nm),^5^ at similar radiation parameters, have been described to yield a reduction of IOP similar to that of ALT. Several advantages characterize LTP, of which, first and foremost, is its ability to lower IOP without relying on patient compliance. Poor patient compliance has been repeatedly shown to be a key challenge in glaucoma medical treatment. There are a myriad of adherence barriers inherent to the glaucoma patient population, such as older age, medication cost, complicated dosing regimens and poor eye drop instillation technique.^6-10^ Noncompliance is especially significant when patients use more than one medication^11^ and ultimately leads to suboptimal, inconsistent IOP control. By replacing or decreasing the need for topical medications, LTP can improve IOP control, reduce systemic side effects such as cardiovascular and respiratory adverse events and depression^12,13^ and local side-effects, such as eyelid dermatitis, lacrimal system scarring, ocular discomfort upon instillation, tear film instability, subconjunctival fibrosis, conjunctival inflammation and epithelium changes and corneal surface and endothelial impairment.^14^

Moreover, by avoiding or delaying the need for filtration surgery, LTP can prevent the numerous and well-recognized associated short-term complications such as hypotony, shallow anterior chamber, bleb leak, and choroidal detachment, as well as long-term complications, such as progression of cataract and life-long risk of endoph-thalmitis.^15,16^

From the financial perspective, LTP seems to be a more cost-effective alternative.^17^

### What is Selective Laser Trabeculoplasty?

In 1983 Anderson and Parrish^18^ discovered that selectively absorbed optical radiation could cause damage (photothermolysis) to a selected pigmented cell population within a tissue composed of multiple cell types. This selective photothermolysis theory, which was first applied in dermatology, made precise aiming unnecessary because the inherent properties of the tissue provided target selectivity. Selective laser trabeculoplasty (SLT) is based on this concept and is possible because pigmented trabecular meshwork (TM) cells exhibit greater optical absorbance of the applied laser energy than the cells that surround them. Therefore, a short burst of laser energy heats and thermally damages pigmented TM cells before neighboring cells have a chance to absorb enough laser energy to incur any thermal damage,^19^ as demonstrated by Latina and Park.^20^

SLT was approved by the FDA in 2002. It uses a 532 nm Q-switched, frequency-doubled Nd:YAG laser which is able to deliver a short pulse of 3 ns duration that limits the conversion of energy to heat. Transmission electron microscopy has demonstrated that SLT results in fracturing of melanin granules and rupturing of lysosomal membranes in the pigmented cells, with the absence of ultrastructural damage in neighboring nonpigmented cells.^20,21^

## MECHANISMS OF ACTION

A number of theories have been proposed regarding the mechanism of LTP:^22^

The main theory attributed to SLT is biological, which suggesting that the decrease in IOP is the result of cellular activity stimulated by the laser energy. Following LTP, there is an increase in the recruitment and number of macrophages in the TM that cause remodeling of the extracellular matrix allowing increased aqueous outflow from the eye.^23^ Other publications^24^ have shown that LTP induces the expression and secretion of both IL-1beta and TNF alpha within the first 8 hours after treatment. These cytokines then mediate increased trabecular stromelysin expression. Putatively, this initiates remodeling of the juxtacanalicular extracellular matrix, a likely site for the aqueous outflow resistance, and thus improves normal outflow facility thereby decreasing IOP. Alvarado^25^ has shown that the number of monocytes/ macrophages in the TM increases substantially after SLT and monocytes augment both outflow facility and Schlemm canal endothelial cell conductivity. He also demonstrated^26^ that SLT, as do prostaglandin analogs (PA), regulates the permeability of cultured human Schlemm's canal cells by inducing intercellular junction disassembly. SLT caused a 3-fold increase in Schlemm's canal cells conductivity which supports the hypothesis that SLT and PA share a common mechanism which mediates their pressure-lowering effects. It also emphasizes the role of intercellular junctions in regulating transendothelial fluid flow across Schlemm canal cells, which are assumed to be the last control point regulating the egress of aqueous humor from the intraocular fluid compartment into the venous compartment^27^ and therefore determining the IOP level. A histopathological study^28^ in human cadaver eyes demonstrated that while the appearance of the areas treated with ALT showed coagulative necrosis of the TM tissue, the areas treated with SLT demonstrated no such evidence of coagulative damage or disruption of the corneoscleral or uveal trabecular beam structure. Rather, it appears to cause cracking of the intracytoplasmic pigment granules and disruption of the trabecular endothelial cells. This study further supports the evidence that the mechanism of action of SLT is biological rather than mechanical.

## CLINICAL TECHNIQUE

SLT uses a frequency doubled, q-switched Nd:YAG laser emitting at 532 nm, with a pulse duration of 3 ns, a spot size of 400 μm and pulse energies ranging from 0.2 to 1.4 mJ, coupled to a slit-lamp delivery system with a He-Ne aiming system.

Several protocols have been evaluated in an attempt to determine the SLT technique with the greatest efficacy.^29^ A comparison between the application of SLT over 90 and 180° using 25 nonoverlapping laser spots per quadrant showed no difference in the pressure response between the two techniques.^30^ Other studies, however, demonstrated greater success rates with 180° and 360° treatments rather than with 90° of SLT application^31^ and better results with 360° SLT than with 180° SLT.^32^ A modified protocol applying 100 overlapping SLT spots over 180° of meshwork led to a poorer response compared with 100 nonoverlapping spots over 360°.^33^

The treatment parameters and technique reported by most authors are the same or very similar to those originally described by Latina.^34^

Treatment with apraclonidine 1%, an alpha-agonist, 1 hour before and just after the laser treatment might prevent a postoperative spike.^35^ Immediately prior to treatment, an application of topical anesthesia is instilled into the eye. The patient is seated at the slit-lamp, a single mirror Gonio lens is used, and the laser is focused on the pigmented TM. Using a 400 μm spot (an area which is 64 times larger than that of the typical 50 μm spot used in ALT) the entire width of the TM is irradiated with each pulse. The laser energy is initially set at 0.8 mJ. If cavitation bubbles (‘champagne bubbles') appear, the energy is reduced in 0.1 mJ increments until there is minimal or no bubble formation and treatment is continued at this energy level. If no cavitation bubbles occur, the energy is increased in increments of 0.1 mJ until bubble formation and then decreased as described above. The entire meshwork is treated with 100 nonoverlapping spots. Some ophthalmologists prefer to limit their initial treatment to180° due to clinical experience with ALT, after which a lower incidence of early IOP rise is known to occur compared with 360° ALT.^35^ Others feel safer with SLT and therefore opt for a more effective procedure, treating 360° in a single session. Postoperatively, steroidal or nonsteroidal anti-inflammatory drops may be prescribed four times a day for 5 to 7 days, although the role of suppressing post-SLT inflammation is unclear especially since, as mentioned previously, cytokine production has been theoretically linked to the IOP-lowering effect of SLT.^36^ Patients usually continue to take their preoperative glaucoma medications until the IOP is re-evaluated.

## INDICATIONS AND CONTRAINDICATIONS

The indications for treatment with SLT are similar to that of ALT:^37^ (i) Newly diagnosed open angle glaucoma (OAG) patients; (ii) OAG patients uncontrolled on medical treatment; (iii) OAG patients with likely or actual poor compliance or poor tolerance to medical treatment; (iv) patients with pseudoexfoliation or pigmentary glaucomas.

It should be noted that IOP elevation after PKP was successfully treated with SLT^38^ which become a valuable therapeutic method that limits invasive surgery for treatment of secondary glaucoma after PKP.

Moreover, IOP elevation after intravitreal triamcinolone acetonide injection may be prevented by performing SLT before the injection^39^ or treated by SLT after the injection.^40^

Current contraindications include: (i) Inflammatory/ uveitic glaucoma, (ii) congenital glaucoma, (iii) poor visualization of the TM.

Contrary to previously held theory, Ho et al^41^ have shown that that IOP is effectively lowered by SLT in eyes with primary angle closure and a patent iridotomy in which there was a sufficient extent of visible TM.

### Worse, Better, Best–SLT in Comparison to Other Modalities

Many comparisons have been made between SLT and other modalities in recent years.

One of the most interesting comparisons was between SLT and ALT.

The Cochrane Database Systematic review of LTP^42,43^ concluded in 2007 that there was some evidence to show similar efficacy in IOP control for SLT and ALT at 6 months and 1 year of follow-up. Since then, multiple retrospective^44^ and prospective^45,46^ clinical trials have been published and found no significant difference in IOP lowering when comparing SLT to ALT^44,47,48^ even over 5 years of follow-up.^49^ However, in retreatment SLT appears to lower IOP more effectively than ALT.^47^

Comparisons have also been made between SLT and medical treatment.

Comparing SLT with latanoprost, Nagar et al reported that SLT decreases pressure in a similar manner to latanoprost. However latanoprost was found to be more likely to reduce IOP fluctuation (success in fluctuation reduction was 50% for SLT and 83% for latanoprost), while SLT had the advantage of being a one-time intervention not requiring ongoing patient compliance.^50^

Comparisons between medication alone and a combination of SLT and medical treatment have shown an additional IOP reduction in patients uncontrolled with medical therapy who were treated with SLT.^34,51-54^ Francis et al^55^ and Klamann et al^56^ were able to reduce the number of glaucoma medications in most of their patients after SLT.

It is generally assumed that SLT treatment is equivalent in IOP lowering to the use of one drug. It must be noted, however, that as a proper dose-response relationship study has not yet been perfomed; it is possible that its effect can be improved.

### ‘Not Destructive – Not Effective?'–SLT Effectiveness

The first efficacy data for SLT was reported by Latina et al^34^ and demonstrated a mean IOP reduction of 6.0 mm Hg (p < 0.001) in eyes previously treated with ALT and 5.8 mm Hg (p < 0.001) in eyes without prior ALT treatment. Overall, 70% of eyes exhibited an IOP reduction of ≥ 3 mm Hg.

Other prospective and retrospective studies^52,57-63^ have reported mean IOP reductions in the range of 3 to 6 mm Hg from pretreatment baselines, equivalent to approximately 15 to 25% reduction from pretreatment IOP with the highest percentage reported being 35.9%^64^ and the lowest 7.9%.^65^

Studies^45,46,65,66^ which have compared the IOP-lowering efficacy of SLT and ALT concluded that SLT and ALT produce statistically equivalent mean IOP reductions, even after 5 years of follow-up.^49^

In a prospective randomized trial conducted by Lai et al^53^ mean IOP reduction after 5 years of follow-up was 8.6 mm Hg (32.1%) in SLT eyes and 8.7 mm Hg (33.2%) in medically treated eyes (p = 0.95). Treatment failure (IOP> 21 mm Hg despite maximal medical therapy requiring filtering surgery) in this study was observed in 17.2% of SLT eyes and 27.6% of medically treated eyes. Other studies^49^ have reported higher failure rate (50%) in a shorter time period ( 2 years). The wide range of success rates in various studies may be explained by the differences in study design and in the many factors which may affect the outcome of SLT including:^62^ glaucoma type, angle status, extent of angle treatment (180 *vs* 360°), pretreatment IOP, number and type of medications and duration of medical treatment before SLT was performed.

SLT has been shown to be effective in almost all glaucoma types: POAG,^42,62,63^ OHT,^67^ NTG,^68^ PXF,^62,69-71^ after cataract surgery,^59^ after intravitreal or subconjunctival triamcinolone,^39,72^ after failed deep sclerectomy,^73^ steroid induced glaucomas^64,74^ and elevated IOP after PKP.^38^

### SLT as Initial Therapy

Nonrandomized, prospective studies by Melamed et al^61^ and McIlraith et al^75^ examined the use of SLT as initial therapy and reported IOP to be reduced by approximately 30% compared to baseline levels, which is comparable with prostaglandin efficacy.^76^ Both trials noted a mean IOP reduction of 8 mm Hg.

A recent, prospective, randomized clinical trial, the SLT/ MED study, compared SLT with various medical treatment regimens as initial treatment and showed that the IOP reduction was similar in both arms after 1 year of follow-up. However, more treatment adjustment steps were needed to maintain target IOP in the medication group.^77^

### Baseline Factors Predictive of SLT Response

In SLT, as in ALT,^78^ higher baseline IOP is almost the only baseline predictive factor of SLT response and found to be highly correlated with greater absolute IOP decrease.^79-81^ No significant differences in IOP response were found with regard to age, phakic status or gender.^79^

The presence of exfoliation, which was previously assumed to have an effect on IOP reduction, was found to have no such effect in 4 months follow-up post-SLT, but was significantly more prevalent in eyes that did not have retreatment, suggesting an association with increased success rate after SLT.^30^ Other publications showed that at 1 year follow-up after SLT, exfoliation glaucoma was not associated with a different outcome compared with OAG ^70,82,83^

TM pigmentation, was also found in most of the studies^79,84,85^ not to affect the success rate of SLT.

Race was also found to have no significant effect in the long-term success rates of SLT (especially with regard to African American and white patients).^20^ Also favorable results have been obtained with SLT in eyes of Asian descent.^53,86-89^

POAG and OHT patients treated with SLT as primary therapy who had thinner corneas (CCT < 555 μm) demonstrated significantly greater percentage of IOP reduction and better IOP control for at least 30 months after SLT.^90^

In pseudophakic patients, SLT response was found to be delayed compared to phakic patients, while the long-term effectiveness (3 months and on) was the same in both groups.^59^

Some reduction in effectiveness was reported in diabetic patients (only 1.2 mm Hg IOP reduction) and in some reports IOP was even higher after SLT.^62,91^

### Adverse Effects of SLT

The most common complication of SLT, as in ALT,^92^ is a transient rise in the IOP, which has been reported in 12% (>10 mm Hg) to 34% (>5 mm Hg) of patients.^31,34,51,93,94^ Generally, these spikes are not associated with any long-term effects and resolve quickly with observation or additional antihypertensive medications.^36^ They have been observed in almost all published series, whether or not the patients were receiving perioperative antihypertensive treatment. In one case series four eyes with a heavily pigmented TM developed markedly elevated IOP following SLT; three of which needed trabeculectomy.^95^

Transient anterior chamber reaction can result from SLT, albeit at a slightly lower rate than ALT.^46^ No significant increase in macular thickness was demonstrated due to this inflammatory reaction.^56^

Other possible side effects, such as redness, pain, and blurred vision, have also been described as transient and without sequelae in all studies.

Transient corneal endothelial changes that have no impact on cell count or visual acuity have also been reported.^96^

Rarely, corneal burns, significant peripheral anterior synechiae, reflux bleeding from the meshwork (Schlemm's) or hyphema have been noted^45,97^ and there is one report of bilateral diffuse lamellar keratitis following consecutive SLT in a LASIK patient.^98^

In summary, it can be said that complications due to SLT are infrequent and their effect is rarely permanent.

### SLT Advantages

In addition to safety, efficacy and independence from compliance considerations, it seems that one of the main advantages of SLT is repeatability. Beneficial IOP reduction with SLT was reported in eyes that had been unresponsive to ALT.^94^ Moreover, Hong et al^99^ demonstrated that in eyes which underwent an initial 360° SLT (first SLT treatment) which was successful for more than 6 months, but eventually lost efficacy and was followed by a second 360° SLT (second SLT treatment), both the first and second treatments significantly reduced the IOP with no significant difference in the efficacy outcomes between the first and second treatments.

Geyer et al^100^ have shown that one 180° SLT treatment in 50 medically uncontrolled eyes facing incisional surgery succeeded in delaying surgery in 66% of the patients at 6 months and 55% of the patients at 12 months; mean IOP reductions were 21 and 20% at 6 and 12 months respectively.

Moreover, as SLT is technically easier to perform compared with ALT, due to the less precise aiming required, it may be used by ophthalmologists with less experience in gonioscopy and angle surgery.

Cost analysis has shown that SLT is ultimately less expensive when compared with the costs of topical medications.^101^ SLT was found to be less costly than latanoprost after 13.1 months,^101^ more cost-effective than 75% adherence PGs, and over 5 years SLT had the lowest total costs when compared to medication or to surgery (p < 0.001).^102^ Taylor^103^ has reported that initial laser trabeculoplasty followed by topical medication and then trabeculectomy was surprisingly cost-effective and was actually cost saving, returning $2.50 for every $1.00 spent, and even if the cost of laser treatment increased 4-fold, it still returned $1.74 for each $1.00 spent.

## SUMMARY

SLT is a safe and effective procedure for reducing IOP. Although its mechanism of action is not fully understood, it provides short and long-term IOP reduction which is consistently equivalent to ALT.

SLT is effective at every stage in the glaucoma treatment algorithm and it may be used as first-line therapy, especially in noncompliant patients or patients who have difficulty taking drops. Similarly, SLT can be used effectively to reduce the number of medications required to control IOP, and can be used in eyes on maximal medical therapy to avoid or delay incisional surgery.

The procedure is easy to perform and well tolerated by patients. Some ophthalmologists prefer an initial treatment of 180° due to previous clinical experience with ALT, but both 180° and 360° treatments have been well studied and both are successful as initial therapy. SLT, is nonmedical so eases concerns over adherence to side effects of, and costs of medical therapy. SLT therefore has a major role to play in the ophthalmologist's armamentarium for reducing IOP.
